# Supplementary material for: Differential Antibody Response against Conformational and Linear Epitopes of the L1 Proteins from Human Papillomavirus Types 16/18 Is Observed in Vaccinated Women or with Uterine Cervical Lesions
Source: Vaccines (Basel). 2021 May 2;9(5):442. doi: 10.3390/vaccines9050442 (PMC8147477; doi:10.3390/vaccines9050442)
Supplement: Supplementary file 1 [file vaccines-09-00442-s001.zip › vaccines-1174151-supplementary.pdf]

Supplementary Table S1

Validation of ELISA system to detect anti-VLPs and anti-L1 antibodies from HPV16/18.

Population group	VLPs Seropositivity		L1 Seropositivity	
	**HPV16**	**HPV18**	**HPV16**	**HPV18**
Vaccinated vs Control [1] ***(n= 36/50)***	**n= 41**	**n= 22**	**n= 33**	**n= 28**
Sensitivity	100.0	61.1	88.9	69.4
Specificity	100.0	98.0	100.0	100.0
Accuracy	100.0	82.6	95.4	87.2
AUC [2]	**1.00**	**0.79**	**0.94**	**0.84**

[1] Control, this is the group of young girls non-sexually active, and presumably HPV-naïve.
[2] *AUC,* Area under the ROC curve.

Supplementary Figure S1

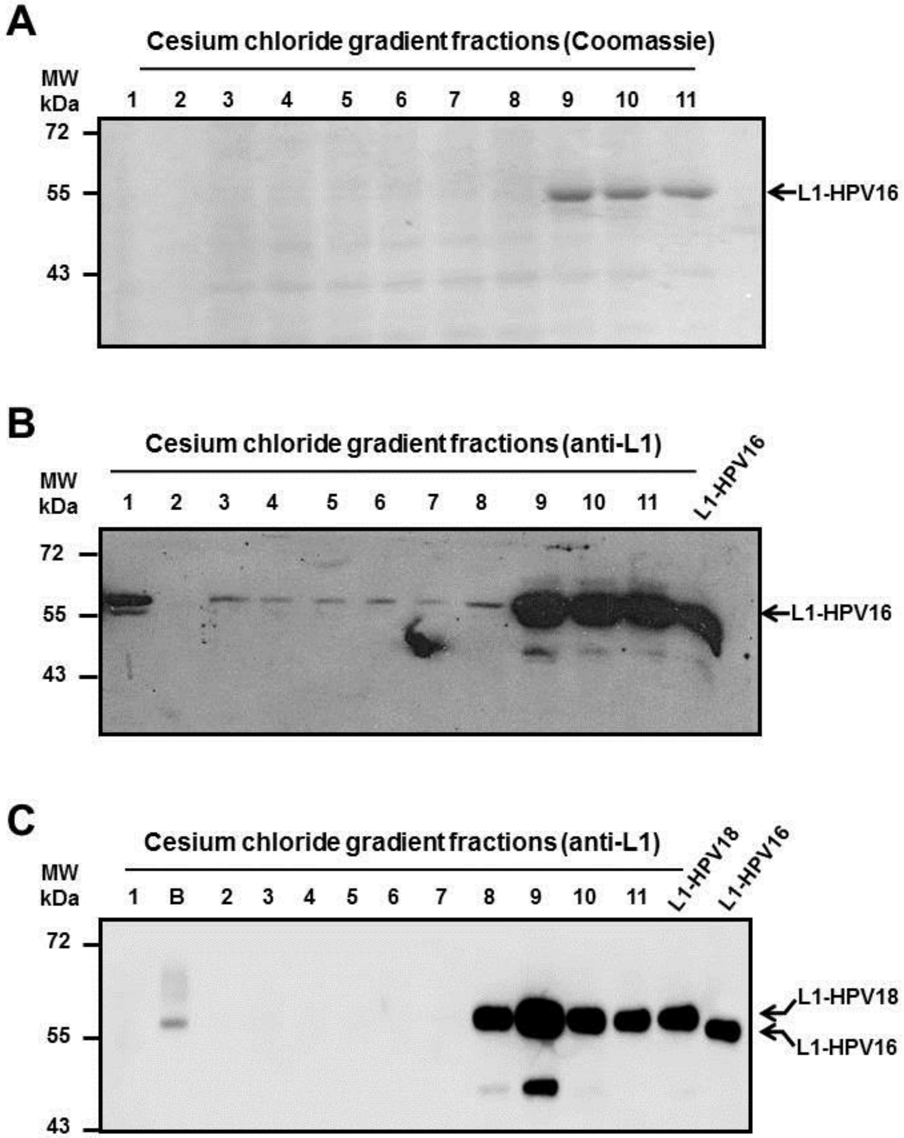

Figure S1. **Identification of HPV16-L1 and HPV18-L1 proteins from CsCl gradient fractions**. (A) Fractions from CsCl gradient of High Five cells infected with BACU-L1-16 were separated on a 10 % acrylamide gel and Coomassie blue stained to visualize protein purity. The L1 proteins were visualized by Western blot in the different gradient fractions using an anti-L1 mouse monoclonal antibody (dil. 1:2000) (DAKO) that recognized the L1 proteins from HPV16 (B) and HPV18 (C). The specific antibody complex was developed by chemiluminescence and membranes exposed to X-OMAT film. Arrows show the specific L1 proteins.

Supplementary Figure S2

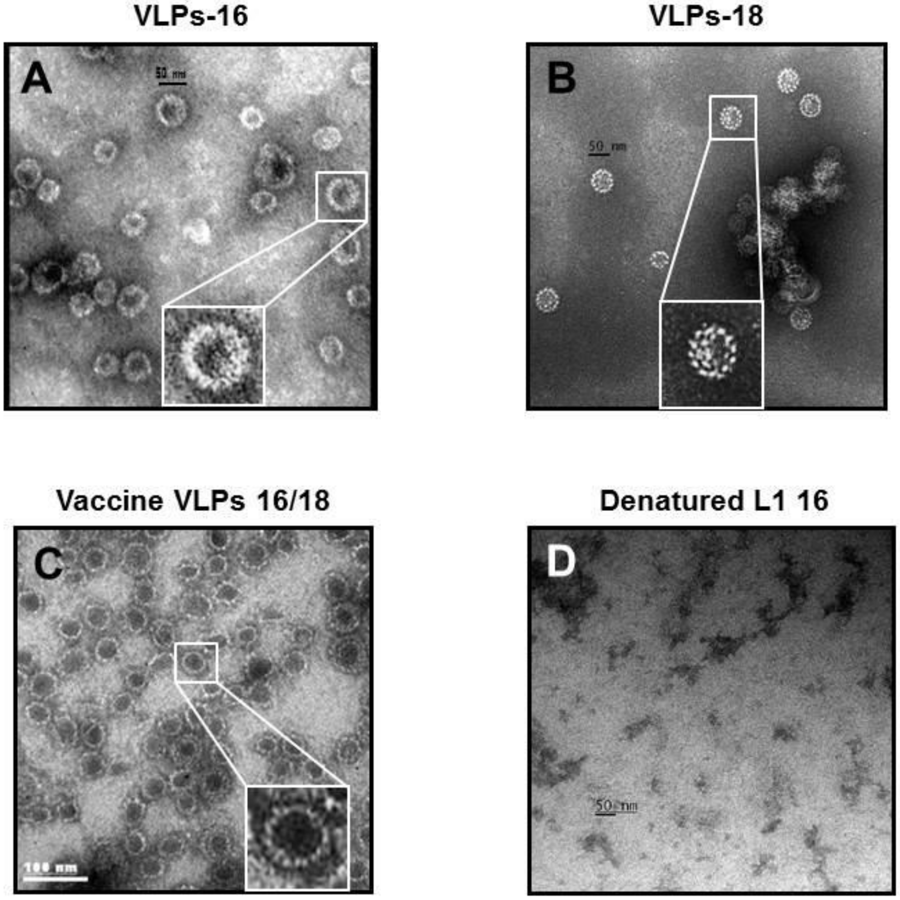

Figure S2. Electron micrographs of VLPs from HPV16/18 produced in baculovirus. Purified CsCl gradient VLPs 16/18 were prepared for TEM as described under Material and Methods and photographed at a magnification of x 25,000 and x 14,000. (A) Purified VLPs from HPV16; (B) VLPs from HPV18; (C) VLPs-16/18 from HPV vaccine were used as a positive control. (D) Denatured L1 was obtained (monomers and aggregates) by treatment of VLPs with carbonate buffer (pH 9.6) for 16 h at 4o C. Scale bars: A, B, and D, 50 nm; C, 100 nm.
